# Limited hiatal dissection versus Dor-fundoplication in laparoscopic Heller myotomy for achalasia: First experience in Morocco - A case control comparison study

**DOI:** 10.1016/j.ijscr.2025.111137

**Published:** 2025-03-11

**Authors:** Abdelmounaim Aitali, Othmane Bourouail, Youssef Elmahdaouy, Abderrahman Elhjouji

**Affiliations:** Visceral Surgery Service II Department, Military Teaching Hospital Mohamed V, Rabat UHC IBN Sina, Rabat, Morocco

**Keywords:** Achalasia, Dysphagia, Laparoscopic Heller myotomy, Limited hiatal dissection, Dor fundoplication, Gastroesophageal reflux disease

## Abstract

**Introduction and importance:**

Laparoscopic Heller myotomy is a primary treatment for achalasia, addressing impaired esophageal motility. Fundoplication is typically added to prevent postoperative reflux. This study compares outcomes of limited hiatal dissection without antireflux system in laparoscopic Heller myotomy to Dor fundoplication.

**Case presentation:**

A retrospective analysis was conducted on 45 patients treated at visceral surgery department (2008–2022). Of these, 29 patients underwent limited hiatal dissection, and 16 underwent Dor fundoplication. A liquid diet was followed on day one, with discharge on day two, and a semi-liquid diet for three weeks. Outcomes included dysphagia resolution, postoperative Eckardt scores <3, and postoperative reflux incidence. The study compared operative and postoperative data between the two groups.

**Clinical discussion:**

The limited hiatal dissection group had a slightly younger mean age (46.97 years) compared to the Dor fundoplication group (51.75 years). The limited hiatal dissection group had a higher proportion of men (58.6 %) while the Dor group had more women (56.3 %). Dysphagia (100 %) and weight loss (68.9 %) were prevalent symptoms. Perioperative complications and hospital stay duration were similar. Operative time was significantly shorter in the limited hiatal dissection group (96.7 vs. 118.3 min, p = 0.004). Both groups showed similar (OR = 0.519, CI = 0.066–4.083) and significant improvement in dysphagia (91.3 % vs. 87.5 %, p < 0.001) with comparable postoperative gastroesophageal disease (20.7 % vs. 25 %, p = 0.726 OR = 1.278, 95 % CI: 0.301–5.420).

**Conclusion:**

Limited hiatal dissection provides comparable symptom relief and reflux prevention, offering a viable alternative to routine antireflux in achalasia treatment.

## Highlights

• Laparoscopic Heller Myotomy provides dysphagia relief in 70% and 90% of achalasia cases.
• Postoperative challenges, including persistent dysphagia and gastroesophageal reflux disease, remain significant concerns.
• Partial fundoplication is effective in reducing gastroesophageal disease but may add complexity and risk persistent dysphagia.
• Limited hiatal dissection approach preserves the physiological anti-reflux system while minimizing complications and reduce operative duration.
• In light of our study, limited hiatal dissection proves to be an equivalent approach to Dor fundoplication, offering comparable efficacy in the alleviation of symptoms and postoperative reflux prevention.

## 1 Introduction

Laparoscopic Heller myotomy (LHM) is the gold standard for achalasia treatment, providing dysphagia relief in over 90 % of cases [1,2]. Achalasia is a motility disorder characterized by impaired esophageal peristalsis and incomplete relaxation of the lower esophageal sphincter, leading to difficulty swallowing and diminished quality of life [2,3]. However, persistent dysphagia and gastroesophageal reflux disease (GERD) remain common postoperative challenges [[4], [5], [6]].

Partial fundoplication, often added to LHM, has been shown to reduce postoperative GERD. Despite its efficacy, the necessity of routine antireflux system (ARS) remains debated due to increased procedural complexity, higher surgical costs, and potential for persistent dysphagia [[7], [8], [9], [10]].

LHM with limited hiatal dissection (LHD) without ARS offers an alternative approach, focusing on preserving the anatomical integrity of the physiological anti-reflux system (PARS) while minimizing complications [11]. This innovative surgical adjustment aims to enhance dysphagia outcomes while concurrently minimizing the risks associated with postoperative reflux complications [12].

The role of fundoplication in LHM continues to be a subject of contention. This study aims to evaluate the outcomes of LHM with LHD compared to those achieved with Dor fundoplication (DF). By analyzing postoperative dysphagia resolution, GERD prevention, and overall effectiveness, it seeks to determine whether LHD without ARS is a viable and effective option in achalasia management.

## 2 Methods

This retrospective study, conducted at the Department of Visceral Surgery, aimed to compare the outcomes of LHM with LHD without routine ARS to the standard LHM technique with anterior DF. Initially, we employed LHM with routine DF. Subsequently, we adopted the approach of LHD without systematic DF and sought to compare the efficacy of this technique with DF procedure. This study was approved by the institutional review board of our surgical department and our hospital, waiving ethical. All procedures in studies involving human participants were performed according to the ethical standards of the institutional research committee and with the 1964 Helsinki declaration and its later amendments or comparable ethical standards. Written informed consent for participation in this case series and accompanying images was obtained from the patient.

The study included patients with achalasia who underwent LHM between 2008 and 2022. Of the total patients, 29 underwent LHM with LHD without ARS, while 16 had LHM with DF. Patients who underwent open surgery or lacked adequate follow-up data were excluded. Data extracted from medical records included demographic information (age, sex), clinical (symptoms, duration of symptoms, peri-operative Eckardt score (ES)), paraclinical (barium esophagogram (BE), high-resolution manometry, 24-h pH monitoring) and operative data.

### 2.1 Surgical procedure

The anterior myotomy with LHD was achieved laparoscopically with no routine DF. This technique was adopted at our service by all surgeon professor with minimal modification for the entire study's interval.

The patient was placed in a dorsal decubitus position with a 30° incline. Pneumoperitoneum was established using either the open technique or Veress needle in the left hypochondrium. Five trocars were positioned: one for the camera (midline periumbilical), one for liver retraction (subxiphoid), two working ports (left and right hypochondrium), and an optional assistant port (left hypochondrium).

**Fig. 1:**
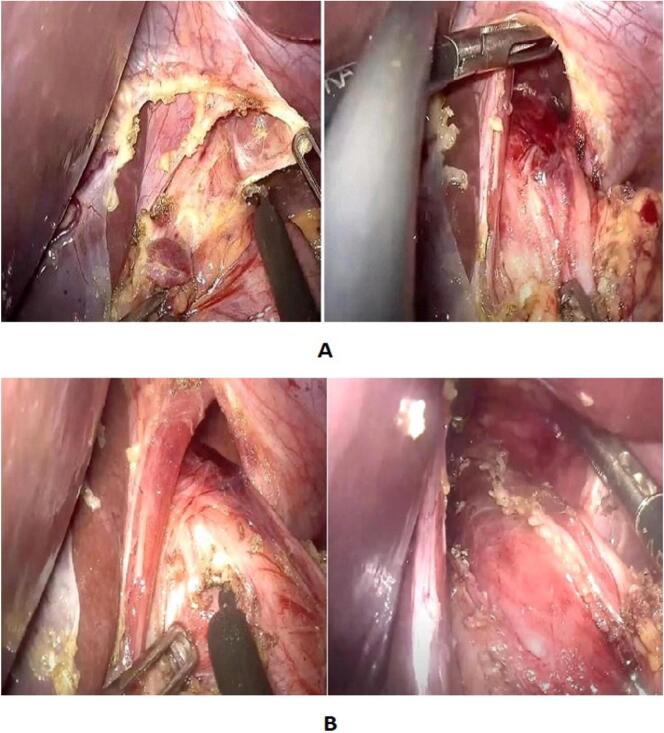
A: Limited hiatal and cardial dissection. B: Anterior eso-cardiomyotomy with monopolar surgical hook.

To expose the cardio-esophageal region, we initiate an incision of the pars flaccida of the lesser omentum, followed by the pars condensa and the phreno-esophageal ligament upper and lower limbs on the right side, and exclusively the upper limbs on the left side, thereby preserving the integrity of the angle of HIS. Subsequent to the dissection and liberation of both the right and left borders of the abdominal and diaphragmatic esophagus, extending over a height of 8 cm from the cardia, the anterior surface and lateral borders of the esophagus are released without perturbing its posterior aspect. This approach allows for the preservation of the meso-esophagus and vascularization of the esophagogastric junction (EGJ). The vagus nerve is identified and safeguarded throughout the procedure. It is noteworthy that if it is troncular, the vagus nerve is reclined towards the right side (Fig. 1A).

The myotomy was initiated 6 cm above the cardia and extended 1 cm below using a monopolar coagulating hook (Fig. 1B). Methylene blue dye was injected via a gastric tube to confirm the esophageal mucosa's integrity. If perforation, fragility, or a hiatal hernia was detected, an antireflux valve (Dor anterior hemivalve) was constructed.

### 2.2 Postoperative follow-up

Postoperative care involved a liquid diet and removal nasogastric tube on Day 1, discharge on Day 2, and semi-liquid diet for twenty one days. Patients were followed at two weeks, one month, three months, six months, and annually thereafter. Evaluations included clinical examinations, ES assessments, and pH monitoring. BE and manometry were performed for symptomatic patients.

### 2.3 Outcomes

The clinical success criteria were defined as follows: resolution of dysphagia and an ES < 3. Postoperative GERD was assessed based on reflux symptoms and esophageal exposure to gastric acidity (EEGA). Pathological exposure was defined as a percentage of time with a pH < 4 exceeding 7 %. Furthermore, ideal benchmarks were set for operative time and hospital stay, with an operative time ≤ 90 min and a hospital stay ≤2 days.

Descriptive results included demographic, clinical, and paraclinical data, followed by operative details.

We evaluated the efficiency of LHM combined with either LHD-ND or DF and compared the two study groups in terms of perioperative complications, operative duration, hospital stay, functional outcomes, and GERD prevention.

### 2.4 Statistical analysis

Data analysis was performed using IBM SPSS Statistics version 26. Descriptive statistics included frequencies, means, and standard deviations. We used parametric and non parametrics test to evaluate and compare the effectiveness between the two study groups. A p-value <0.05 was considered statistically significant. Literature searches were conducted on platforms including PUBMED, Google Scholar, EM Consulted, and ScienceDirect.

Our case series was meticulously prepared following the updated 2023 PROCESS guidelines, ensuring adherence to internationally recognized standards for the reporting of surgical case series [13].

## 3 Results

The study encompassed 45 patients who underwent LHM, categorized into two groups, 29 patients (64.4 %) in the limited hiatal dissection with no-Dor fundoplication (LHD-ND) group and 16 patients (35.6 %) in the Dor fundoplication (DF) group. The mean age was marginally lower in the LHD-ND group, at 46.97 ± 11.3 years, compared to 51.75 ± 11.2 years in the DF group, with an overall mean of 48.67 ± 11.3 years. Concerning gender distribution, men constituted 58.6 % and women 41.4 % of the LHD-ND group, whereas the DF group demonstrated a reversed pattern, with men representing 43.8 % and women accounting for 56.3 %.

All patients presented with dysphagia (100 %), which was the predominant symptom, while, the weight loss, emerging as a secondary concern, affected 31 patients (68.9 %), including 20 (69 %) in the LHD-ND group and 11 (68.8 %) in the DF group. Regurgitation was observed in 21 patients (46.7 %), distributed as 13 (44.8 %) in the LHD-ND group and 8 (50 %) in the DF group. Additionally, reflux was reported by 15 patients (33.3 %), with 10 (34.5 %) in the LHD-ND group and 5 (31.3 %) in the DF group. Chest pain, another notable symptom, was present in 17 patients (37.8 %), with 10 (34.5 %) in the LHD-ND group and 7 (43.8 %) in the DF group. The average duration of symptoms was 42.76 ± 28 months in the LHD-ND group, 42.31 ± 29.3 months in the DF group, and 42.6 ± 28.1 months overall. Similarly, the mean follow-up period was 39.6 ± 11.2 months in the LHD-ND group, 38.2 ± 6.8 months in the DF group, and 38.9 ± 9.4 months across all patients.

Abnormalities were observed in 84.4 % of cases during BE studies, revealing dilation and/or stenosis. According to the Ressano-Machaealli classification [14], Stage II was the most frequent finding in both study groups, with 11 cases (37.9 %) in the LHD-ND group and 8 cases (50 %) in the DF group, for a total of 19 cases (42.2 %). The mean integrated residual pressure over 4 s (IRP-4S) was 24.43 ± 7.8 in the LHD-ND group, 26.9 ± 4.6 in the DF group, and 25.3 ± 6.9 across all patients. Based on the Chicago classification [15], type II achalasia was the most prevalent, accounting for 60 % of cases, with 18 patients (62.1 %) in the LHD-ND group and 9 patients (56.3 %) in the DF group. Additionally, endoscopy identified gastritis in 8 patients (17.8 %), including 5 in the LHD-ND group and 3 in the DF group.

The mean length of myotomy was 7.17 ± 0.53 cm in the LHD-ND group and 7.19 ± 0.4 cm in the DF group, with an overall mean of 7.18 ± 0.49 cm. Intraoperative complications (11.1 %) included perforation in 3.4 % of the LHD-ND group and 6.3 % of the DF group, bleeding was declared in 3.4 % of the LHD-ND group and 6.3 % of the DF group, when, subcutaneous thoracic emphysema was observed only in the DF group (6.3 %). Postoperative fistulas occurred only in the LHD-ND group (3.4 %), while, conversion presented in 6,7 % of patients. Regarding the mean procedure duration, a significant difference was noted, with 96.72 ± 8.5 min in the LHD-ND group compared to 118.3 ± 15 min in the DF group (+21.58 min, p < 0.001), indicating that the procedure was significantly longer in the DF group. The mean hospital stay was 2.51 ± 1.9 days in the LHD-ND group and 2.56 ± 1.4 days in the DF group (Table 1).

**Table 1:** Demographic, symptoms, paraclinical examination and operative data for the two study groups of achalasia (LHD no- Dor (ND) group and Dor-fundoplication (DF) group).

	LHD no-Dor Gp N (%)	Dor-fundoplication Gp N (%)	Total N (%)
*Demography, symptoms, paraclinical exams*			
N patients	29 (64.4 %)	16 (35.6 %)	45
Average age (years)	46.97 ± 11.3	51.75 ± 11.2	48.67 ± 11.38
Man	17 (58.6 %)	7 (43.8 %)	24 (53.3 %)
Woman	12 (41.4 %)	9 (56.3 %)	21 (46.7 %)
Dysphagia	29 (100 %)	16 (100 %)	45 (100 %)
Weight loss	20 (69 %)	11 (68.8 %)	31 (68.9 %)
Regurgitation	13 (44.8 %)	8 (50 %)	21 (46.7 %)
Reflux	10 (34.5 %)	5(31.3 %)	15 (33.3 %)
Chest pain	10 (34.5 %)	7 (43.8 %)	17 (37.8 %)
Mean symptoms duration (months)	42.76 ± 27.9	44.78 ± 38.3	43,27 ± 30.14
Average follow-up (months)	39.6 ± 9,2	38.2 ± 7,8	38.9 ± 11,4
Barium esophagogram - stage			
1	5 (17.2 %)	5 (31.3 %)	10 (22.2 %)
2	11 (37.9 %)	8 (50 %)	19 (42.2 %)
3	4 (13.7 %)	3 (18.8 %)	7 (15.6 %)
4	2 (6.9 %)	0	2 (4.4 %)
NA	3 (10.3 %)	0	3 (6.7 %)
Non-dilated	4 (13.8 %)	0	4 (8.9 %)
IPR-4S mmgh (mean)	24.43 ± 7.8	26.93 ± 4.64	25.32 ± 6.9
Chicago_classification			
1	8 (27.6 %)	5 (31.3 %)	13 (28.9 %)
2	18 (62 %)	9 (56.3 %)	27 (60 %)
3	3 (10.4 %)	2 (12.5 %)	5 (11.1 %)
Endoscopy: gastritis	5 (17.3 %)	3 (18.8 %)	8 (17.8 %)

*Operative parameters*			
The average length of the myotomy (cm)	7.17 ± 0.53	7.19 ± 0.4	7.18 ± 0.49
Perforation	1 (3.4 %)	1 (6.3 %)	2 (4.4 %)
Hemorrhage	1 (3.4 %)	1 (6.3 %)	2 (4.4 %)
Subcutaneous thoracic emphysema	0	1 (6.3 %)	1 (2.2 %)
Postoperative fistula	1 (3.4 %)	0	1 (2.2 %)
Conversion	1 (3.4 %)	2 (12.5 %)	3 (6.7 %)
Average procedure duration (min)	96.72 ± 8.49	118.31 ± 15	104.58 ± 15
Average hospital stay (days)	2.51 ± 1.9	2.56 ± 1.4	2.53 ± 1.75

Dysphagia was initially present in all patients (100 %) but showed a significant improvement postoperatively, decreasing to 6.9 % in the LHD-ND group and 12.5 % in the DF group, with an overall postoperative prevalence of 8.9 % (LHD-ND: P = 0.000; DF: P = 0.000; Total: P < 0.001).

Preoperative weight loss, observed in 69 % of patients in the LHD-ND group and 68.8 % in the DF group, was markedly reduced following surgery, reaching 17.2 % and 25 %, respectively (LHD-ND: P = 0.000; DF: P = 0.016; Total: P = 0.04). Similarly, regurgitation exhibited a substantial decline, from 44.8 % to 10.3 % in the LHD-ND group and from 50 % to 18.8 % in the DF group, leading to an overall rate of 13.3 % (LHD-ND: P = 0.000; DF: P = 0.063; Total: P = 0.007).

**Fig. 2:**
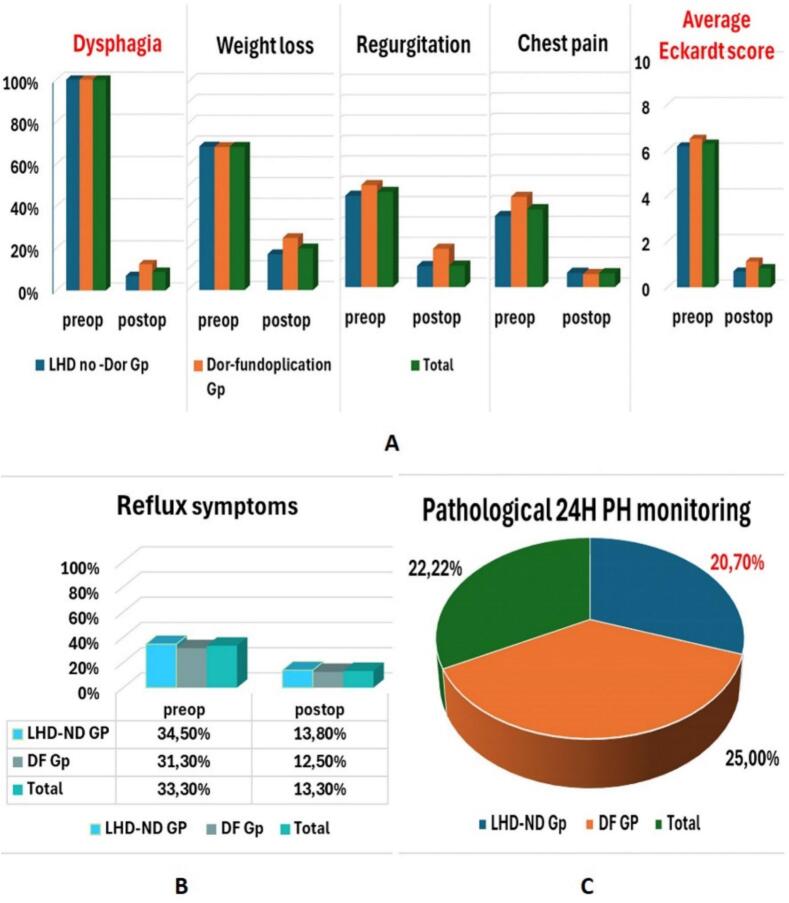
This figure illustrates the functional outcomes and the GERD incidence in the two groups of study (LHD-ND vs DF). A: Functional outcomes in the two groups of study (LHD-ND vs DF) after LHM. B: Graph depicts the improvement in reflux symptoms following LHM in the two study groups. C: Diagram illustrates the incidence of esophageal acid exposure after LHM in the study groups.

Chest pain also significantly decreased in both groups, from 31.5 % to 6.9 % in the LHD-ND group and from 43 % to 6.3 % in the DF group (LHD-ND: P = 0.016; DF: P = 0.031; Total: P = 0.039). The mean Eckardt score demonstrated a marked improvement, decreasing from 6.17 ± 1.03 to 0.69 ± 1.07 in the LHD-ND group and from 6.5 ± 0.8 to 1.13 ± 1.2 in the DF group, with an overall reduction from 6.29 ± 0.96 to 0.84 ± 1.1 (P < 0.001) (Fig. 2A; Table 2).

**Table 2:** Functional outcomes and the incidence of GERD in the study groups (LHD-ND group vs DF group).

Clinical outcomes	LHD-ND (LND) Gp		DF Gp		Total		P value
	Preop	Postop	Preop	Postop	Preop	Postop	
Dysphagia	29 (100 %)	2 (6.9 %)	16 (100 %)	2 (12.5 %)	45 (100 %)	4 (8.9 %)	LHD-ND: 0.000 DF: 0.000 Total: <0.001
Weight loss	20 (69 %)	5 (17.2 %)	11 (68.8 %)	4 (25 %)	31 (68.9 %)	9 (20 %)	LHD-ND: 0.000 DF: 0.016 Total: 0.040
Regurgitation	13 (44.8 %)	3 (10.3 %)	8 (50 %)	3 (18.8 %)	21 (46.7 %)	6 (13.3 %)	LHD-ND: 0.000 DF: 0.063 Total 0.007
Chest pain	9 (31.5 %)	2 (6.9 %)	7 (43.8 %)	1 (6.3 %)	16 (35.6 %)	3 (6.7 %)	LHD-ND: 0.016 DF: 0.031 Total 0.039
Average Eckardt score	6.17 ± 1.03	0.69 ± 1.07	6.5 ± 0.8	1.13 ± 01.2	6.29 ± 0.96	0.84 ± 1.1	LHD-ND: 0.001 DF: <0.001 Total: <0.001
Reflux symptom	10 (34.5 %)	4 (13.8 %)	5 (31.3 %)	2 (12.5 %)	15 (33.3 %)	6 (13.3 %)	LHD-ND: 0.031 DF: 0.375 Total 0.012
Postoperative 24-hour pH monitoring							
N%	29 (100 %)		14 (87.75 %)		43 (92.2 %)		–
Esophageal acid exposure	6 (20.7 %)		4 (25 %)		10 (22.2 %)		–

Regarding gastroesophageal reflux symptoms, a significant reduction was observed in the LHD-ND group, with prevalence decreasing from 34.5 % to 13.8 % (P = 0.031). In contrast, the decrease in the DF group, from 31.3 % to 12.5 %, was not statistically significant (P = 0.375), resulting in an overall reduction from 33.3 % to 13.3 % (P = 0.012).

Despite these symptomatic improvements, 24-h pH monitoring revealed pathological esophageal acid exposure in 20.7 % of patients in the LHD-ND group and 25 % in the DF group, with an overall prevalence of 22.2 % (Fig. 2B and C; Table 2).

The operative data and postoperative outcomes of LHM were compared between the LHD-ND group and the DF group. According to the matching test for the two study groups, none of the selected variables (age, sex, clinical data, preoperative Eckardt score, paraclinical data) has a significant influence on group affiliation.

In terms of perioperative complications, the rate was 10.3 % in the LHD-ND group and 18.7 % in the DF group, with no statistically significant difference (p = 0.650; OR = 2; 95 % CI: 0.353–11.318). The conversion rate, which was more prevalent in the DF group (12.5 % vs. 3.4 %), also did not show a significant difference (p = 0.285; OR = 4; 95 % CI: 0.333–47.990). A notable distinction was observed regarding operative duration, with 34.5 % of procedures in the LHD-ND group completed in ≤90 min, compared to only 6.25 % in the DF group (p = 0.067; OR = 0.127; 95 % CI: 0.115–1.103). This suggests that an optimal operative duration of 90 min may be marginally significant in the LHD-ND group, although the odds ratio indicates that this likelihood is reduced. A larger sample size may be required for a more definitive conclusion. Finally, the length of hospital stay was comparable between the two groups, with 89.7 % of patients in the LHD-ND group and 86.7 % in the DF group being discharged within 2 days, with no significant difference (p = 0.906; OR = 1.112; 95 % CI: 0.182–6.906).

The analysis of postoperative outcomes reveals an excellent improvement in the relief of dysphagia, with a slightly higher rate observed in the LHD-ND group compared to the DF group (93.1 % vs. 87.5 %). However, the difference between the two groups was not statistically significant (p = 0.608, OR = 0.519, 95 % CI = 0.066–4.083). Regarding the ES <3, 93.1 % of patients in the LHD-ND group achieved this threshold, compared to 87.5 % in the DF group. Once again, the difference was not significant (p = 0.608, OR = 1.92, 95 % CI = 0.245–15.185). With respect to reflux symptoms, the incidence was similar between the two groups (13.8 % vs. 12.5 %), and no statistically significant difference was observed (p = 0.903, OR = 1.120, 95 % CI: 0.182–6.906). Finally, EEGA, also showed no significant difference between the groups (p = 0.726, OR = 1.278, 95 % CI: 0.301–5.420) (Table 3).

**Table 3:** Comparative study of operative data and functional outcomes after LHM in the two groups of study.

Operative datas, postoperative outcomes	LHD-ND Gp	DF Gp	p-Value	OR	95 % CI
Peri-operative complication	10.3 %	18,8 %	0.650	2	0.353–11.318
Conversion	3.4 %	12,5 %	0.285	4	0.333–47.990
Operative duration ≤ 90 min	34.5 %	6.25 %	0.067	0.127	0.115–1.103
Hospital stay ≤ 2 days	89.7 %	86.7 %	0.906	1.112	0.182–6.906
Relief from dysphagia	93.1 %	87.5 %	0.608	0.519	0.066–4.083
Eckardt score (ES) < 3	93.1 %	87.5 %	0.608	1.92	0.245–15.185
Reflux symptoms	13.8 %	12.5 %	0.903	1.120	0.182–6.906
Pathological 24 hour PH monitoring	20.7 %	25 %	0.726	1.278	0.301–5.420

## 4 Discussion

Achalasia, characterized by inadequate relaxation of the lower esophageal sphincter during swallowing, presents a challenge in finding effective and lasting treatments [9]. Current medical or surgical interventions, such as pneumatic dilatation or botulinum toxin injections, often offer only temporary relief for dysphagia. In contrast, LHM stands out as a durable solution, showing success rates ranging from 77 % to 100 % in various series with a 3-year average follow-up [1,16]. While LHM is effective, the potential for post-surgery GERD is a concern [2,8]. To address this, surgeons commonly recommend incorporating ARS with LHM, making it more suitable than peroral endoscopic myotomy (POEM) [17,18]. Among fundoplication techniques, the anterior 180° DF is recognized as the preferred choice [[19], [20], [21]]. However, automatically adding ARS to LHM raises questions, as that is not the primary objective of this surgery [11,12,22]. Previous randomized controlled trials have explored its potential in reducing EEGA, providing conflicting findings on its necessity [8]. Meanwhile, numerous studies cast doubt on its requirement [11,23,24].

The use or non-use of an ARS during LHM has sparked much debate within the medical community. It is crucial to adopt a balanced approach to optimize clinical outcomes while minimizing adverse effects associated with this procedure. In this context, we wish to reopen the debate on the necessity of fundoplication. Our study advocates for a refined approach—LHD in LHM without routine ARS, this technique carefully mobilizes the anterior and lateral esophagus while conserving anatomical structures, particularly the meso-esophagus and HIS angle. By adhering to this procedure, we aim to maintain the vascularization of the EGJ and contribute to the overall preservation of PARS anatomy and function [11,23]. The speculation arises that maintaining this anatomical mechanism through controlled cardio-esophageal dissection could significantly reduce postoperative EEGA [12]. Our results were significant, achieving a 96,6 % success rate in relieving dysphagia, with a similar GERD incidence of 17,2 % that reported in literature.

Several authors advocate incorporating anterior partial fundoplication with LHM to prevent fibrous retraction and GERD [25,26]. This recommendation is supported in numerous studies, highlighting a significantly lower incidence of pathological GERD (31.5 % vs. 8.8 %, P = 0.003), while maintaining comparable dysphagia control [18,27,28]. A randomized double-blinded clinical trial demonstrates the effectiveness of adding Dor-fundoplication in preventing EEGA after LHM for achalasia. The trial shows a significantly higher median EEGA time (4.9 %) in the LHM alone group compared to the LHM with Dor-fundoplication group (0.4 %). Moreover, postoperative lower esophageal sphincter (LES) pressures did not differ, and dysphagia resolution was comparable in both groups [16].

Despite these findings, some authors contest the necessity of an ARS after LHM, suggesting it may lead to dysphagia recurrence due to the absence of peristalsis [29]. The Vanderbilt Centre supports LHM without ARS, prioritizing maximum relief from dysphagia while minimizing disruptions to the angle of His, with the goal of reducing postoperative gastroesophageal reflux [30]. In alternative laparoscopic studies, comparisons of abnormal pH-manometry findings between groups with and without DF revealed statistically non-significant rates. Lyas et al. reported rates of 7.9 % and 10 %, respectively [6], while Santoro et al. documented 21.3 % and 22.9 % in the two groups undergoing LHM alone versus LHM with ARS, respectively, with slightly higher dysphagia incidence [31]. Furthermore, Bloomston's study concluded that the postoperative need for anti-acid treatment was 13 % among patients who underwent LHM alone, compared to 10 % in those who received additional DF [9]. Zaninotto et al. observed an 8.8 % recurrence of dysphagia after LHM with DF, compared to our results in which only 2.8 % presented recurrence of dysphagia [4,8].

Three similar studies in the literature have analyzed outcomes of LHD with and without DF, as well as complete hiatal dissection (CHD) with and without DF. Ramacciato et al. compared two groups of 32 patients, the first underwent LHM without ARS (15 patients) and the second had DF (17 patients). Results showed a similar clinical success rate between the two groups, with a slight advantage for the LHM without Dor group (100 %) compared to the LHM with Dor group (94 %). However, this favorable difference in functional outcomes was accompanied by a higher incidence of GERD in the LHM alone group (20 %) versus 5.8 % in the LHM with Dor group [8]. Simic's study compared three groups: LHM with LHD without DF (G1), LHM with LHD and Dor (G2) and LHM with complete hiatal dissection (CHD) and Dor (G3). Results revealed that the G3 group had a much higher reflux rate, with a DeMeester score of 23.1 %, compared to 8.5 % in G2, and 9.1 % in G1. This suggests that LHD, with or without DF, better protects against reflux than CHD. Overwise, the addition of Dor-technique had no significant impact on postoperative GERD outcomes in LHD (9.1 % vs 8.5 %) [12]. Finally, DeHaan's [11] study compared LHD with no ARS and CHD with Dor procedure in a sample of 31 patients. After 24 months of follow-up, achalasia severity questionnaire (ASQ) and Gastroesophageal reflux disease health-related quality of life (GERD-HRQL) questionnaire scores showed similar results in both groups, suggesting equivalent management of symptoms and reflux [11]. However, this similarity raises questions about the validity of the questionnaires used. It might have been more relevant to explore other assessment methods, such as pH monitoring and endoscopy, for a more objective analysis (Table 4). As demonstrated in our study, these studies suggest a similar observation, while the LHD no-Dor group shows better relief of dysphagia, both groups exhibit comparable results in terms of GERD control.

**Table 4:** Summary of three retrospective studies, analyzing the outcomes of limited hiatal dissection (LHD) with and without Dor-fundoplication, as well as complete hiatal dissection (CHD).

Authors, year of publication	Study type	N patients		Average follow-up (months)	Clinical success		Postoperative GERD	
		LHM-LHD no-Dor	LHM-Dor		LHM-LHD no-Dor	LHM-Dor	LHM-LHD no-Dor	LHM-Dor
Ramacciato,2005	Retrospective	15	17	6	RD 100 %	RD: 94 %	EEGA: 20 %	EEGA: 5.8 %
Petar Simić, 2009	Retrospective	G1 (22 cases)	LHD G2 (36 cases) vs CHD G3 (26 cases)	36	↗Average pressure of LES G1, G2 versus G3		EEGA: 9.1 %	EEGA 8.5 (LHD) vs 23.1 % (CHD)
Reece DeHaan, 2016	Retrospective	10	CHD [21]	24	ASQ: similar		GERD-HRQL: similar	
Our study, 2024	Retrospective	29	9	36	ES < 3 and RD 93.1 %	ES < 3: and RD 87.5 %	EEGA: 20.7 %	EEGA: 25 %

LHM: laparoscopic Heller myotomy. LHD: limited hiatal dissection. CHD: complete hiatal dissection.

LES: lower esophageal sphincter. RD: relief of dysphagia. ASQ: Achalasia Severity Questionnaire.

ES: Eckardt score. EEGA: esophageal exposure to gastric acid. GERD-HRQL: gastroesophageal reflux disease health-related quality of life.

The key question we have highlighted pertains to the enduring GERD encountered by patients undergoing LHM and DF. This is particularly noteworthy as it represents a departure from established surgical norms [11,12,26]. In his 2001 series, Mr. Richard opposed the regular use of ARS, contending that both total and partial fundoplication increase resistance to flow across the LES, diminishing symptom relief [30]. Individuals with achalasia inherently face susceptibility to prolonged EEGA postoperatively, and the introduction of ARS amplifies this vulnerability [30]. This hypothesis is supported by Kumar et al., who reported a low incidence (6 %) of endoscopically confirmed esophagitis following LHM with LHD without partial ARS [23].

In comparison with other studies involving LHM with DF, the LHD-ND group in our series demonstrated a dysphagia improvement rate of 93.1 %, which is comparable to the outcomes reported by Sze Li Siow (100 %), Ali Sürmelioğlu (100 %), and Eriksson (91.6 %). Similarly, control of GERD was consistent across these studies, with rates of 20.8 %, 21.8 %, and 30.2 %, respectively. Conversely, the dysphagia relief rate observed in studies by Zaninotto and Emmanuel Asti was slightly lower (87 %) compared to our series, yet these authors reported superior control of GERD (6 % versus 20.7 % in our study) [27,[32], [33], [34], [35]]. Balancing optimal dysphagia relief with effective GERD prevention remains a significant challenge in the surgical management of achalasia. In this context, a comparison with earlier studies involving LHM and DF suggests that the LHD approach without ARS offers promising results, highlighting its potential and warranting further investigation.

This prompts a reassessment of the necessity of incorporating ARS, balancing cost-effective surgical approaches and potential risks associated with recurrent dysphagia [11,12,36]. While post-myotomy acid reflux can be managed through medication, introducing fundoplication to control reflux may inadvertently heighten dysphagia, potentially requiring more aggressive interventions like pneumatic dilatation or reoperation [31]. A randomized clinical trial of 43 patients evaluated the long-term cost-effectiveness of LHM with DF compared to LHM alone in patients with achalasia. The data showed that the initial surgical cost per patient was higher for LHM with Dor ($942 more, p = 0.04) due to a longer operative time (+40 min, p = 0.01). However, when considering proton pump inhibitor treatment costs, LHM + Dor became less costly after 3.2 years, and the cost difference widened over time. At 10 years, LHM with Dor was associated with a total cost of $6861 and a quality-adjusted life expectancy of 9.9 years, compared to $9541 and 9.5 years for LHM alone [10]. However, this study warrants further critique, as it did not consider the impact of LHD.

Acknowledging inherent constraints, our retrospective study's limitations stem from potential variability despite comprehensive patient information efforts. The less cases of LHM with DF limits comparisons. Regardless of these limitations, our study's strength lies in dedicatedly exploring the benefits of LHD in Heller myotomy without routine ARS, providing nuanced insights and reviving the discussion on regularly incorporating fundoplication in LHM, particularly when minimal dissection of esophago-cardial area allows preserving the PARS's integrity.

This nuanced exploration sheds light on multifaceted considerations surrounding the choice of ARS, urging a thoughtful approach to optimize both patient outcomes and healthcare costs. The practice of LHD, foundational to POEM, not only addresses these concerns but also offers additional advantages, this approach facilitates prompt responses to potential intraoperative complications, particularly enabling the utilization of the ARS when the cardio-esophageal mucosa is perforated or deemed fragile [12,37]. Proposals for long-term studies form a key part of the discussion, especially in assessing outcomes related to LHD no-fundoplication versus adding standard ARS. The need for additional investigations, including 24 h-pH studies and postoperative esophageal clearance, are underlined to determine the appropriateness of ARS in specific cases.

## 5 Conclusion

In our study, the limited hiatal dissection approach in laparoscopic Heller myotomy demonstrated reduced operative duration while maintaining equivalent safety and efficacy to the Dor fundoplication procedure, highlighting its potential as a viable alternative.

## Abbreviations

LHM: Laparoscopic Heller myotomy
GERD: Gastroesophageal reflux disease
ARS: Antireflux system
LHD: Limited hiatal dissection
PARS: Physiological anti-reflux system
EGJ: Esophagogastric junction
BE: Barium esophagogram
POEM: Peroral endoscopic myotomy
LES: Lower esophageal sphincter
LHD-ND: Limited hiatal dissection no-Dor
DF: Dor fundoplication
EEGA: Esophageal exposure to gastric acid
ES: Eckardt score

## Statement of informed consent

Written informed consent for publication of this case series and accompanying images was obtained from the patient. A copy of the written consent is available for review by the Editor-in Chief of this journal on request.

## CRediT authorship contribution statement

Aitali A – Methodology, Formal analysis, Writing – review & editing, Validation
Bourouail O – Conceptualization, Methodology, Data curation, Data analysis, Writing – original draft
ELmahdaouy Y – Data curation, Investigation
Elhjouji A – Formal analysis, Writing – review & editing.

## Ethical approval

This study was approved by the institutional review board of Visceral Surgery department, waiving ethical approval. All procedures in studies involving human participants were performed according to the ethical standards of the institutional research committee and with the 1964 Helsinki declaration and its later amendments or comparable ethical standards.

## Guarantor

Othmane Bourouail.

## Research registration number

Not applicable.

## Funding

No funding was provided for the completion of this manuscript.

## Declaration of competing interest

The authors declare that they have no competing interests.

## Data Availability

The datasets used during the current study available from the corresponding author on reasonable request.
